# Preserving Pure Siamese Crocodile Populations: A Comprehensive Approach Using Multi-Genetic Tools

**DOI:** 10.3390/biology12111428

**Published:** 2023-11-13

**Authors:** Thitipong Panthum, Nattakan Ariyaraphong, Wongsathit Wongloet, Pish Wattanadilokchatkun, Nararat Laopichienpong, Ryan Rasoarahona, Worapong Singchat, Syed Farhan Ahmad, Ekaphan Kraichak, Narongrit Muangmai, Prateep Duengkae, Yusuke Fukuda, Sam Banks, Yosapong Temsiripong, Tariq Ezaz, Kornsorn Srikulnath

**Affiliations:** 1 Animal Genomics and Bioresource Research Unit (AGB Research Unit), Faculty of Science, Kasetsart University, 50 Ngamwongwan, Chatuchak, Bangkok 10900, Thailand; thitipong.pa@ku.th (T.P.); nattakan.ari@ku.th (N.A.); gamewongsathit@gmail.com (W.W.); pish.wa@ku.th (P.W.); nararat.l@ku.th (N.L.); rasoarahonarivoniaina.h@ku.th (R.R.); worapong.singc@ku.ac.th (W.S.); syedfarhan.ah@ku.ac.th (S.F.A.); ekaphan.k@ku.th (E.K.); ffisnrm@ku.ac.th (N.M.); prateep.du@ku.ac.th (P.D.); 2 Special Research Unit for Wildlife Genomics (SRUWG), Department of Forest Biology, Faculty of Forestry, Kasetsart University, 50 Ngamwongwan, Chatuchak, Bangkok 10900, Thailand; 3 Department of Botany, Kasetsart University, 50 Ngamwongwan, Bangkok 10900, Thailand; 4 Department of Fishery Biology, Faculty of Fisheries, Kasetsart University, 50 Ngamwongwan, Bangkok 10900, Thailand; 5 Department of Environment, Parks and Water Security, Northern Territory Government, Darwin, NT 0830, Australia; yusuke.fukuda@nt.gov.au; 6 Research Institute for the Environment and Livelihoods, College of Engineering, IT and the Environment, Charles Darwin University, Darwin, NT 0909, Australia; sam.banks@cdu.edu.au; 7 R&D Center, Sriracha Moda Co., Ltd., Sriracha, Chonburi 20230, Thailand; yosapong@srirachamoda.com; 8 Centre for Conservation Ecology and Genomics, Institute for Applied Ecology, Faculty of Science and Technology, University of Canberra, Bruce, ACT 2617, Australia; tariq.ezaz@canberra.edu.au; 9 Laboratory of Animal Cytogenetics and Comparative Genomics (ACCG), Department of Genetics, Faculty of Science, Kasetsart University, 50 Ngamwongwan, Bangkok 10900, Thailand

**Keywords:** conservation, hybrid, reintroduction, saltwater crocodile, Siamese crocodile, SNP

## Abstract

**Simple Summary:**

Hybrids between the critically endangered Siamese crocodile (*Crocodylus siamensis*) and least-concern saltwater crocodile (*C. porosus*) pose a significant challenge to conservation efforts due to human activities. Previous studies used microsatellite and mitochondrial DNA data to identify these hybrids, but potential biases and genetic drift within the populations could influence the results. To address these limitations and gain a clearer understanding of the genetic dynamics, we employed DArT sequencing to identify genome-wide single nucleotide polymorphisms (SNPs) in both species and confirm hybrid scenarios. We compared a population of Australian saltwater crocodiles to assess species-specific SNP distribution. Various analytical approaches were used to diagnose hybridization levels, especially in cases with potential backcrossing. Approximately 17.00–26.00% of loci were shared between Siamese and saltwater crocodile genomes. We identified 8051 species-specific SNP loci for Siamese crocodiles and 1288 for saltwater crocodiles. Using a PCR-based approach, three SNP loci were developed as markers, successfully enabling the distinction between species and various levels of hybridization. By combining mitochondrial and nuclear genetic information with species-diagnostic DNA markers, we developed a novel method for conservation prioritization, aiding long-term species survival through reintroduction and management programs.

**Abstract:**

Hybrids between the critically endangered Siamese crocodile (*Crocodylus siamensis*) and least-concern saltwater crocodile (*C. porosus*) in captive populations represent a serious challenge for conservation and reintroduction programs due to the impact of anthropogenic activities. A previous study used microsatellite and mitochondrial DNA data to establish the criteria for identifying species and their hybrids; however, the results may have been influenced by biased allelic frequencies and genetic drift within the examined population. To overcome these limitations and identify the true signals of selection, alternative DNA markers and a diverse set of populations should be employed. Therefore, this study used DArT sequencing to identify genome-wide single nucleotide polymorphisms (SNPs) in both species and confirm the genetic scenario of the parental species and their hybrids. A population of saltwater crocodiles from Australia was used to compare the distribution of species-diagnostic SNPs. Different analytical approaches were compared to diagnose the level of hybridization when an admixture was present, wherein three individuals had potential backcrossing. Approximately 17.00–26.00% of loci were conserved between the Siamese and saltwater crocodile genomes. Species-diagnostic SNP loci for Siamese and saltwater crocodiles were identified as 8051 loci and 1288 loci, respectively. To validate the species-diagnostic SNP loci, a PCR-based approach was used by selecting 20 SNP loci for PCR primer design, among which 3 loci were successfully able to differentiate the actual species and different hybridization levels. Mitochondrial and nuclear genetic information, including microsatellite genotyping and species-diagnostic DNA markers, were combined as a novel method that can compensate for the limitations of each method. This method enables conservation prioritization before release into the wild, thereby ensuring sustainable genetic integrity for long-term species survival through reintroduction and management programs.

## 1. Introduction

Hybridization is a fascinating topic in evolutionary biology and a common occurrence in nature, and it represents a valuable source of genetic diversity by allowing species to acquire beneficial traits and adaptations, thereby contributing to lineage divergence and speciation [1]. In contrast, anthropogenic hybrids pose a significant threat to the genetic purity and survival of a species, particularly when they arise due to conservation management [2]. These hybrids often resemble their parental species, which increases the risk of introgression in reintroduction programs, such as those for crocodiles (*Crocodylus* spp.) [3,4]. Interspecific hybridization has been observed in crocodile lineages, even though different chromosome numbers occur among species [5]. Studying the consequences of introgression systems can enhance the role of hybridization in shaping biodiversity and clarify the evolutionary relationships within species. Hybridization between sympatric species, such as Morelet’s crocodile (*Crocodylus moreletti*) and the American crocodile (*C. acutus*), has been widely reported as a serious issue in wild crocodile populations [6]. Moreover, anthropogenic impacts have led to the formation of hybrids between Siamese (*C. siamensis*) and Cuban crocodiles (*C. rhombifer*) or Siamese and saltwater crocodiles (*C. porosus*) [3,4], which represents a serious problem for conservation programs because Siamese crocodiles are critically endangered species and saltwater crocodiles are classified as least concern [7]. The large genetic admixture in hybrids between Siamese and saltwater crocodiles and their functions remain poorly understood [3,4], despite the potential threat of hybridization to some populations of endangered crocodilians.

The Siamese crocodile has a restricted distribution in Thailand, Cambodia, Vietnam, and Laos, whereas the saltwater crocodile is a generalist species found in India, Southeast Asia, Northern Australia, and the Pacific Islands [8]. Despite their different adaptations and distributions, both crocodile species are important for conservation. Genomic data from both mitochondrial and nuclear microsatellite markers have been used to estimate the divergence time and genetic distance between Siamese and saltwater crocodiles [3,4,9] These two species likely diverged from a common ancestor approximately 20 million years ago and have since been genetically isolated [5]. However, reciprocal hybridization between Siamese and saltwater crocodiles has been observed in captivity. F_1_ hybrids and backcross (BC) crocodiles are fertile and have been reported to grow faster than either parental species [10]. Hybridization and introgression threaten the genomic integrity of captive Siamese crocodile populations, which poses a challenge to conservation efforts because Thai crocodile farms, which are members of the Thai Crocodile Farm Association (TCFA), are willing to donate Siamese crocodiles for reintroduction [4]. In our previous studies [3,4], the genetic structure and population history of Siamese and saltwater crocodiles in Thailand were elucidated by combining mitochondrial DNA (mtDNA) and microsatellite data. The results showed significant genetic differentiation between the populations, suggesting the existence of multiple distinct evolutionary lineages or subpopulations within the species. The hybrid individuals in the sampled population may have been associated with a limited number of microsatellite loci, which cannot cover the entire genome [3,4]. In addition, the allelic frequency and gene pool may have been biased by the specific population analyzed, and the influence of genetic drift could have caused a loss of genetic diversity [11]. To overcome these limitations, alternative DNA markers should be employed, and a diverse set of populations should be added to identify the true signals of selection that span across divergent populations.

Here, we aimed to confirm the genetic scenario of the hybrids identified in our previous study [3,4] and reveal the genetic relationship between the two crocodile species. Genome-wide single nucleotide polymorphisms (SNPs) were identified using DArT sequencing in both species. Recent genotyping-by-sequencing (GBS) through technologies like Diversity Arrays Technology (DArTseq) by Diversity Arrays Technology, Pty Ltd., is an effective method for identifying species-diagnostic loci using SNP loci and generating restriction site-specific presence/absence (PA) markers in non-model species [12]. This method is accomplished by combining restriction enzymes to isolate low-copy sequences (most informative for marker discovery and typing) from the repetitive portion of the genome. Other GBS technologies, such as RADseq, utilize only one enzyme with various internal technical procedures and still retain more repetitive fractions [13]. A population of saltwater crocodiles from Northern Australia was used to compare the distribution of species-diagnostic SNPs because the historical distribution of Siamese crocodiles was narrower than that of the broader range of saltwater crocodiles. The saltwater crocodile population in Australia is within the distribution range, but it is thought to be significantly larger than what saltwater crocodiles from Thailand can migrate to. This makes it a crucial resource for the crocodile species’ gene pool. This study also aimed to characterize the genomic features of a unique crocodile hybridization system. Based on the results of this study, the following key questions were explored: (i) Can the evolutionary relationships between and within these crocodile species be clarified? (ii) Can parental and hybrid individuals be differentiated into distinct lineages? This information has practical implications for conservation management decisions in “the Siam Crocodile Bioresource Project” [4].

## 2. Materials and Methods

### 2.1. Specimen Collection and DNA Extraction

Specimens of Siamese and saltwater crocodiles from Thai captive populations were collected with permission from farm owners and the TCFA, as previously described by [3]. Saltwater crocodile specimens from the Australian population were collected in Darwin, Australia, in the previous study [14], and the same individuals were used. Detailed information on the sampled individuals is presented in Table S1. Animal care and experimental procedures were approved by the Animal Experiment Committee of Kasetsart University, Thailand (approval no. ACKU04959). The study protocol was also approved by the Animal Ethics Committee of the Australian University (Animal Ethics Protocol Number A2017/11) and the Parks and Wildlife Commission of the Northern Territory (Research Permit Number 7902), Department of Parks and Wildlife of the Western Australian Government (Permit Nos. 08-002527-1 and 08-002527-2), and Parks Australia of the Australian Government (Permit No. AU-COM2017-361). All procedures were conducted in accordance with the Regulations on Animal Experiments at Kasetsart University. Blood samples were collected from the ventral tail vein using a 23-gauge needle attached to a 2 mL disposable syringe containing 10 mM ethylenediaminetetraacetic acid for DNA extraction. Whole-genome DNA extraction was performed following a standard salting-out protocol. Each DNA sample was evaluated by gel electrophoresis in the presence of high molecular-weight DNA and then stored at −20 °C until required for DArTseq library construction.

### 2.2. Development of DArT Arrays

The DArTseq method was based on Jaccoud et al. [15], with the genotyping of multiple loci performed using DArTseq™ (Diversity Arrays Technology Pty Ltd., Canberra, Australia) for SNP loci and in silico DArT for presence/absence (PA) loci to assess the genetic diversity, population structure, and species-diagnostic SNPs. DArTseq™ arrays were developed using approximately 100 ng of DNA from each specimen. DNA was then digested and ligated as previously described [16]. Sequences were processed using proprietary DArTseq analytical pipelines [17]. DArTsoft14 outputs were filtered using criteria such as the reproducibility value (>3.5), alternate allele (>0.9), and call rate (>0.8; proportion of samples with markers scored) [12]. To mitigate genotype calling bias, the output was filtered using sequencing depth criteria, creating three distinct datasets with depths of >5X, >10X, and >15X.

### 2.3. Genotyping Assay and Marker Selection

For SNP data, “0” referred to the reference homozygote, “1” to the heterozygote, and “2” to the alternate allele homozygote, and “-” was assigned to the double null/null allele homozygote. PA data were coded as “1” for presence, “0” for absence, or “-” for putative heterozygosity. The reference data for this study consisted of the whole-genome sequence of the saltwater crocodile (accession no. GCA_001723895.1). Loci with alternate allele homozygosity and reference allele homozygosity with a frequency of at least 100% and heterozygosity lower than or equal to 0% were retained for Siamese crocodile markers, and they differed from those of the saltwater crocodile. For saltwater crocodile markers, loci differing from those of the Siamese crocodile and showing alternate allele homozygosity and reference allele homozygosity with a frequency of at least 100% and heterozygosity lower than or equal to 0% were retained. These loci were compared with those of hybrid crocodiles for species identification. The number of combined markers between Siamese crocodile and saltwater crocodile individuals for pairwise differences among SNP and PA loci was determined by performing a Hamming distance calculation using the “rdist” function of R version 4.1.2 statistical software. Heatmaps were plotted using the ggplot2 R package [18]. The number of pairwise differences between all individuals across all loci is represented by the Hamming distance. The Cochran–Armitage trend test (CATT) was conducted using the “catt” function of R version 4.1.2 with the HapEstXXR package to investigate the genetic association between each locus and phenotypic data from SNP and PA loci. The CATT results were comparable to those of the chi-square test, which examined whether the distribution of various genotypes adhered to the null hypothesis. The informativeness of the SNP and PA loci was evaluated using a polymorphic information content (*PIC_in_*) index. *PIC* values ranging from zero (fixation of one allele) to 0.5 (equal frequencies of both alleles) were calculated for each locus. The parameters were calculated for various datasets, including sequencing depths of >5X, >10X, and >15X.

### 2.4. Genetic Diversity and Population Structure Analyses

Genetic analyses involved the pooling of the SNP and PA loci, with both groups assigned to the SNP loci. The parameters were calculated for multiple datasets, encompassing sequencing depths exceeding 5X, 10X, and 15X. Allelic frequency, number of alleles (*N*_a_), effective number of alleles (*N*_e_), observed heterozygosity (*H*_o_), and expected heterozygosity (*H*_e_) were calculated for pairwise comparisons of Siamese–saltwater crocodiles, Siamese (CSI)–saltwater (CPOTH) crocodiles (Thai population), and Siamese (CSI)–saltwater (CPOAU) crocodiles (Australian population). Welch’s *t*-test, which does not assume equal variance between samples, was used to test for significant differences between *H*_o_ and *H*_e_ across samples. The “t.test” function of the “stats” package in R version 4.1.2 was used for this purpose [18]. Deviations from the Hardy–Weinberg equilibrium, Shannon’s information index (*I*), and the fixation index (*F*) were calculated for each locus of the population. Relatedness values (*r*) were calculated using GenAlEx version 6.5 [19] based on allelic frequencies in the population, and the mean pairwise r values were obtained [19]. The same approach was adopted to compare the inbreeding coefficients (*F*_IS_) between populations and the overall *F*_IS_. Wright’s F-statistic for genetic differentiation between populations (*F*_ST_) was calculated using GenAlEx version 6.5.

The population structure between Siamese and saltwater crocodiles was determined using FastSTRUCTURE through the “gl2faststructure” function in R [18]. The software estimated the number of hypothetical subpopulations (*K*) using a Bayesian clustering approach that groups genetically similar genotypes into the same subgroups. To visualize the overall relationship across individuals in the populations, a principal coordinate analysis (PCoA) was conducted using the “pcoa” function in the “ape” package, while a discriminant analysis of principal components (DAPC) was performed using the ADEGENET 2.0 package in R 4.1.2 [18,20]. No assumptions were made regarding the population models in the DAPC method [21]. Additionally, a dendrogram was created using the R package ‘ape’ and by performing a cluster analysis with the unweighted pair-group method (UPGMA) and grouping the data accordingly [21]. A tree-based approach was utilized in the TreeMix software v1.13 to reconstruct historical relationships among the populations under analysis [22].

### 2.5. Species Delimitation

A species-level examination was performed based on two single-loci datasets between the Siamese and saltwater crocodiles, which were tested using the General Mixed Yule Coalescent (GMYC) [23] and Bayesian implementation (BI) of the Poisson tree processes (bPTP) methods [24]. For the GMYC delimitation method, an ultrametric tree was constructed in BEAST v2.0.2 [25], which relied on the uncorrelated lognormal relaxed clock GTR + I + G and a coalescent tree prior. The MCMC was run for 20 million generations, and trees and parameters were sampled every 1000 generations. Log files were visualized in Tracer v1.5 [26] to assess the stationary state of the parameters to estimate the effective sample size (ESS). After removing 25% of the trees as burn-in, the remainder was used to generate a single summarized tree in TreeAnnotator v2.0.2 (part of the BEAST v2.0.2 package) as an input file for the GMYC analyses with a single-threshold model using the function “gmyc” in the R package “splits” (R-Forge, http://r-forge.r-project.org/projects/splits/, accessed on 20 June 2023). For the bPTP method, the non-ultrametric consensus tree obtained through the above-mentioned BI analyses was used for species delimitation, which was performed by running 50,000 MCMC generations on an online server (http://species.h-its.org/, accessed on 20 June 2023), with all other parameters set to default.

### 2.6. Homology Searching

SNP loci meeting our criteria and demonstrating statistically significant associations with species-diagnostic loci were subjected to a BLAST search of the NCBI against the reference genomes of the saltwater crocodile, American alligator (*Alligator mississippi*), false gharial (*Tomistoma schlegelii*), gharial (*Gavialis gangeticus*), and chicken (*Gallus gallus*). The BLASTn program was used to search all loci in the NCBI database (http://blast.ncbi.nlm.nih.gov/Blast.cgi, accessed on 20 June 2023). The search considered only sequences with E-values lower than 0.001 and query coverage with similarities greater than 70%.

### 2.7. Functional Annotation and Gene Ontology of Siamese and Saltwater Crocodiles

Functional annotation was performed to understand the biological functions of the species-diagnostic SNP loci. Reference annotation of the saltwater crocodile gene dataset was used to perform a BLASTn search of all candidate loci [27]. A reference gene dataset was retrieved from the Ensembl database using the Biomart package v.110 (Ensembl Genome Server, 2022). Significant hits with an identity of >95% and alignment length of >65 bp were retained from the tabular-formatted output file generated from the BLASTn results. Gene sequences corresponding to significant hits were extracted from the reference dataset and mapped using NCBI-BLASTx against the saltwater crocodile proteome dataset, which included all annotated proteins [28]. The proteome dataset was downloaded from UniProtKB/Swiss-Prot [29]. UniProtKB is a protein database that provides comprehensive and reliable information on protein functions through accurate, consistent, and detailed annotations. Functional annotations and Gene Ontology (GO) enrichment analyses were also conducted on the filtered gene hits using ShinyGO (0.76) implemented in the R/Bioconductor packages. The best-matching species genome was used as a reference in the analysis, with standard settings that included a 0.05 FDR *p*-value threshold [30]. Crocodile genes associated with GO terms and biological pathways were retrieved using Blast2GO v2.0.36 [31]. Associated GO terms describing biological processes (BPs), molecular functions (MFs), and cellular components (CCs) were detected by processing the matching transcripts. GO categories were identified using UniProtKB, the Gramene Protein Database (GR_protein), and the Protein Data Bank (PDB).

### 2.8. Identification of Conserved and Species-Diagnostic SNPs

The SNP files of Siamese and saltwater crocodiles were imported using datasets and alignments. The consensus section of the sequence viewer options was then enabled, with “consensus” selected as the “disagreement” option. The output included nucleotide statistics, such as identical sites (%) and pairwise identity (%), and the values were obtained using Geneious Prime v2023.0.1.

### 2.9. Validation Assays

Primers were designed based on the position of species-diagnostic SNPs in both Siamese and saltwater crocodiles. Sufficient 5 bp (TGCAG) flanking sequences on either side of the SNP loci were identified from the saltwater crocodile reference genome and then filtered according to four filtering criteria: (a) no repeated sequences, (b) SNP-free within the flanking sequence, (c) one of two alleles in accordance with the base in the reference, and (d) flanking sequence in both Siamese and saltwater crocodiles ≥50 bp (Table 1). PCR amplification was performed using 20 μL of 10X buffer, containing 1.5 mM MgCl_2_, 0.2 mM dNTPs, 0.5 μM primers, 0.5 U *Taq* polymerase (Apsalagen Co. Ltd., Bangkok, Thailand), and 25 ng genomic DNA. The PCR conditions were as follows: an initial denaturation at 94 °C for 3 min, followed by 10 cycles of 94 °C for 30 s, 70 °C for 30 s, and 72 °C for 30 s, followed by 10 cycles of 94 °C for 30 s, 67 °C for 30 s, and 72 °C for 30 s, followed by 15 cycles of 94 °C for 30 s, 65 °C for 30 s, and 72 °C for 30 s, and a final extension at 72 °C for 5 min. The PCR products were examined by electrophoresis on 1% agarose gels. The partial mtDNA D-loop region was employed as a positive PCR control marker using the primers described by Lapbenjakul et al. [3] to verify that the absence of PCR products in males was not caused by PCR failure. In addition to the crocodile specimens mentioned above, 115 crocodile specimens from our previous study [4] were used for DNA marker validation.

## 3. Results

### 3.1. Genotyping Variation, Inbreeding Coefficient, and Relatedness

**Figure 1:**
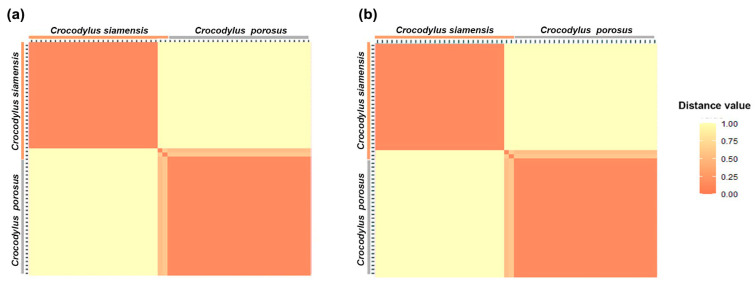
Hamming distance between the Siamese crocodile (*Crocodylus siamensis*, CSI) and saltwater crocodile (*C. porosus*, CPO) at sequencing depths of >5X. (**a**) SNP loci with the criterion of species-diagnostic loci (CSI:CPO) and (**b**) PA loci with the criterion of species-diagnostic loci (CSI:CPO).

A total of 53,160 SNP loci from the saltwater crocodile genome (used as the reference genome) and 28,775 PA loci were sequenced. The *PIC_in_* values ranged from 0.49 to 0.50 for both the SNP and PA markers. The overall distribution of *PIC_in_* values was asymmetrical and skewed towards higher values. At sequencing depths of >5X, a total of 5091 SNP and 4248 PA loci were identified as species-diagnostic loci by applying the criterion of 100:0 Siamese crocodile: saltwater crocodile (CSI:CPO) during filtering. The proportional pairwise Hamming distance between Siamese and saltwater crocodiles using species-diagnostic SNP and PA loci (under the null exclusion model) revealed lower within-species distances of 0.520 ± 0.043 and 0.388 ± 0.029 for SNP and PA loci in Siamese crocodiles, respectively, compared with that of saltwater crocodiles (0.710 ± 0.020 and 0.667 ± 0.026, respectively) (Figure 1 and Figure S1). By contrast, higher between-species distances of 1.000 ± 0.000 for both SNP and PA loci were observed for Siamese crocodiles. A significant locus association with species phenotype was verified for 5091 SNP loci by the CATT test (χ^2^ = 56.00–118.00, *p* < 0.001). For both Siamese and saltwater crocodiles, 4248 PA loci were homozygous for the reference allele in one species and homozygous for the SNP in another species (χ^2^ = 56.00–59.00, *p* < 0.001). For the dataset with sequencing depths exceeding 10X and 15X, all values exhibited similarity and followed the same trend, as shown in Table S2.

SNP and PA loci were pooled for the genetic analyses, with both groups assigned to the total SNP (SNP_t_) loci. At sequencing depths of >5X, positive *F* values were observed in the CSI, CPOTH, and CPOAU populations. The *PIC* of all populations ranged from 0.000 to 0.120, and the *I* value ranged from 0.000 to 0.217. The *H*_o_ values ranged from 0.000 to 0.020 (mean ± standard error [SE]: 0.002 ± 0.000), and the *H*_e_ values ranged from 0.000 to 0.079 (mean ± SE: 0.046 ± 0.001) (Table 2). A significant difference was observed between *H*_o_ and *H*_e_ using Welch’s *t*-test (Table 3). A comparison of the pairwise *H*_o_ values between populations revealed statistical differences between two pairs (CSI-CPOTH and CPOTH-CPOAU), whereas the pairwise *H*_e_ values showed differences between one pair (CSI-CPOTH) (Table 4). The overall mean *F*_IS_ was 0.887 ± 0.001, whereas the mean *F*_IS_ values of CSI, CPOTH, and CPOAU were 0.816 ± 0.206, 0.843 ± 0.031, and 0.896 ± 0.018, respectively. The overall mean relatedness value (*r*) was −0.037, and the mean relatedness values of CSI, CPOTH, and CPOAU were −0.035 ± 0.036 (−0.971–0.071), −0.043 ± 0.024 (−0.478–0.043), and −0.035 ± 0.030 (−0.517–0.098), respectively. Significant differences (*p* < 0.05) were observed in the estimates of *F*_ST_ between all crocodile populations after 110 permutations (Table S3). Statistical differences were observed for CSI-CPOTH and CPOTH-CPOAU. AMOVA revealed that 89% of the genetic variation was within populations, whereas 10% was between populations. In the dataset characterized by sequencing depths surpassing 10X and 15X, similarity and a consistent trend were observed, as shown in Tables S4–S6.

### 3.2. Clustering of Siamese and Saltwater Crocodile Populations

**Figure 2:**
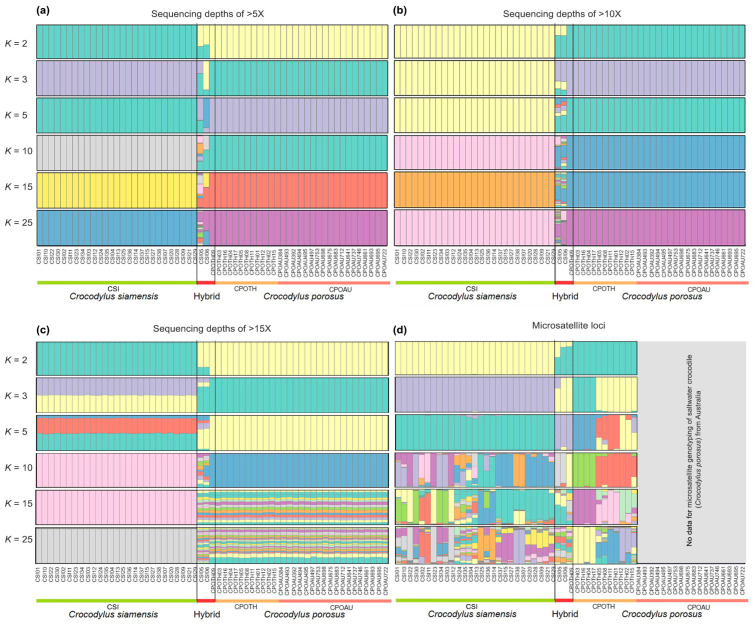
Structure of filtered data of SNP loci (**a**) sequencing depths of >5X, (**b**) sequencing depths of >10X, and (**c**) sequencing depths of >15X among Siamese crocodile (*Crocodylus siamensis*), saltwater crocodile (*Crocodylus porosus*), and hybrid crocodile. (**d**) Structure of microsatellite genotyping for Siamese and saltwater crocodiles, as well as the hybrids derived from the previous studies [3,4].

The model-based Bayesian clustering algorithms implemented in STRUCTURE generated different population patterns (with three different datasets of sequencing depth >5X, >10X, and >15X) with increasing *K*-values of 2, 3, 5, 10, 15, and 25; however, the optimized population structure patterns were assigned a *K*-value of 3 based on Evanno’s Δ*K* (Figure 2). Saltwater crocodiles from Thailand and Australia clustered in the same group.

The PCoA revealed that the first, second, and third principal components accounted for 95.45%, 3.54%, and 0.96% of the total variation, respectively, and supported three tentatively differentiated crocodile groups (CSI, CPOTH, and CPOAU) (Figure S2a). This was consistent with the DAPC results (Figure S2b). The TreeMix results further supported a separation between time points and indicated hybrid individuals (CSI05 and CSI06) that occurred in the CSITH stands (Figure S3). The resulting UPGMA tree indicated that all Siamese and saltwater crocodiles belonged to their respective species. The two hybrid individuals (CSI05 and CSI06) did not belong to the Siamese crocodile clade. However, the hybrid Individual (CPO09) was identified as a saltwater crocodile based on the UPGMA tree (Figure S4).

### 3.3. Species Delimitation

**Figure 3:**
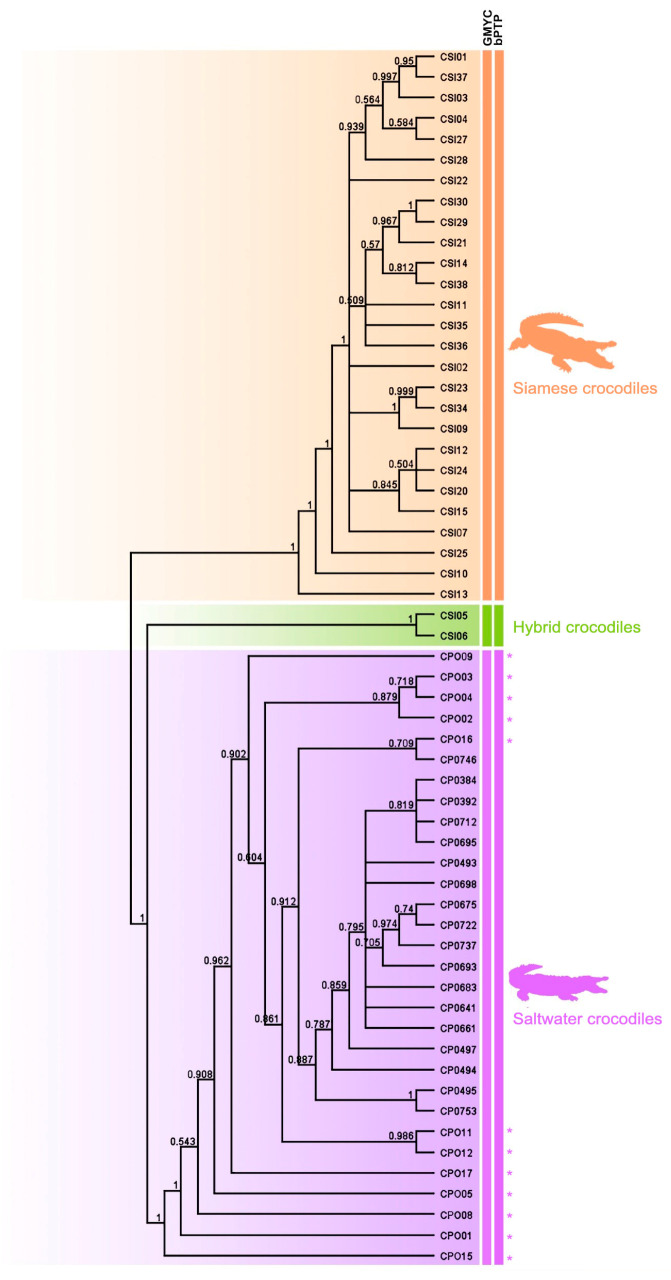
Cladogram clarifying the phylogenetic relationships among the 59 individuals, constructed from a Bayesian inference analysis using genome-wide SNPs. The asterisk (*) is saltwater crocodile (*Crocodylus porosus*) from Thailand.

Using three different datasets of sequence depth, species were delimited between Siamese and saltwater crocodiles using the GMYC and bPTP methods, which showed that all Siamese and saltwater crocodiles were classified into the same respective species. The two hybrid individuals (CSI05 and CSI06) were not identified as Siamese crocodiles using a species delimitation approach. The saltwater crocodile group included one hybrid individual (CPO09), which was previously identified as a hybrid by microsatellite genotyping [3] (Figure 3).

### 3.4. Identification of Conserved and Species-Diagnostic SNPs

At sequencing depths of >5X, of the 9339 SNP_t_ loci, Siamese and saltwater crocodiles shared 25.8% identical SNP_t_ loci. Within the Siamese crocodile population, 79.8% of the SNP_t_ loci were identical, whereas, within saltwater crocodiles from both the Thai and Australian populations, 78.3% of the SNP_t_ loci were conserved. In the dataset with sequencing depths exceeding 10X and 15X, similarity was observed among all values, and they conformed to the same trend, as demonstrated in Table S7.

### 3.5. Homology of Putative Species-Diagnostic Loci

Based on sequencing depths of >5X, the species-diagnostic SNP_t_ loci of Siamese and saltwater crocodiles shared sequence homology with several other species, including the American alligator, false gharial, gharial, and chicken. In Siamese crocodiles, 2033 out of 4534 SNP_t_ loci were identified by global BLAST analyses using NCBI databases, and they were primarily associated with the short hematopoietin receptor family 2, conserved site, DNA repair metallo-beta-lactamase, tenascin, EGF-like domain, and other pathways. In saltwater crocodiles, however, 264 of the 557 SNP_t_ loci were primarily associated with the methyltransferase small domain, mixed (including eukaryotic/archaeal prmc-related), peptide chain release factor, mixed (including granulin), and iron-binding zinc finger cdgsh type pathways (Figure S5).

### 3.6. Functional Classification and Enrichment Analysis of Siamese and Saltwater Crocodile Loci

**Figure 4:**
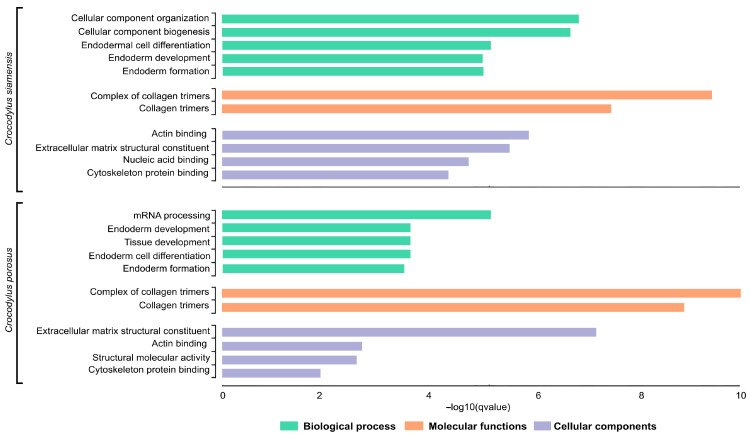
Gene Ontology (GO) functional classification of Siamese crocodile (*Crocodylus siamensis*) and saltwater crocodile (*Crocodylus porosus*). Histograms of the frequency of transcripts annotated to specific GO categories; biological processess, molecular functions, and cellular components are represented by green, orange, and purple bars, respectively.

Species-diagnostic SNP loci in Siamese and saltwater crocodiles were subjected to GO enrichment analyses. For the Siamese crocodile, the species-diagnostic SNP_t_ loci were enriched in the BP terms cellular component organization, cellular component biogenesis, endodermal cell differentiation, and endoderm formation, the MF terms collagen trimers and their complexes, and the CC terms included extracellular matrix structural constituent, actin binding, cytoskeleton protein binding, and nucleic acid binding. For the saltwater crocodile, the species-diagnostic SNP loci were enriched in the BP terms mRNA processing, endoderm and tissue development, and cell differentiation, the MF terms collagen trimers and their complexes, and the CC terms extracellular matrix structural constituent, actin binding, structural molecular activity, and cytoskeleton protein binding (Figure 4).

### 3.7. Validation of Species-Diagnostic Loci with DNA Markers

**Figure 5:**
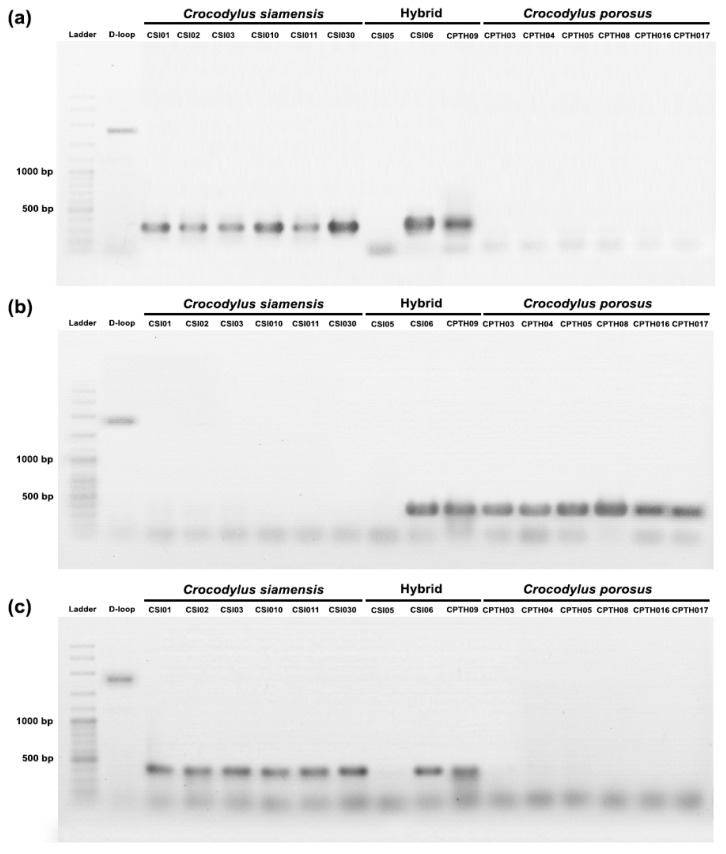
PCR validation species-diagnostic loci among Siamese crocodile (*Crocodylus siamensis*), saltwater crocodile (*Crocodylus porosus*), and hybrid crocodile. (**a**) primer CST09 (locus id: 23111606), (**b**) primer CST01 (locus id: 23113297), and (**c**) primer CPT08 (locus id: 34606790).

A PCR-based method was used to validate species-diagnostic loci in Siamese and saltwater crocodiles. DNA markers were randomly developed from twenty loci, and three of them (locus id: 23111606, 34606790, and 23113297) were partially validated (Table 1). Two primer pairs (primer CST09 and primer CST08), which were developed from locus id: 23111606 and 34606790, produced 320 bp and 450 bp DNA bands, respectively, in Siamese crocodiles and hybrid individuals (CSI06 and CPO09) (Figure 5). Another primer pair (primer CST01) was developed from locus id: 23113297, and it produced a 400 bp DNA band in saltwater crocodiles and hybrid individuals (CSI06 and CPO09). However, species-diagnostic DNA markers were not amplified in one hybrid (CSI05).

## 4. Discussion

Siamese crocodiles have experienced a population decline as a result of habitat loss and illegal collection in Thailand [32]. Moreover, although the TCFA does not support artificial hybridization, hybridization with saltwater crocodiles has compromised the genetic purity and integrity of Siamese crocodiles in commercial crocodile farms in Thailand [4]. Identifying outbreeding depression in hybrids from parental species prior to reintroduction is crucial for avoiding the risks of conservation programs. Early studies were hindered by the difficulty of detecting DNA variations, which resulted from limited sampling of Thailand [3,4]. With the development of high-throughput genotyping methods, such as SNP assays, DNA markers have become essential for assessing genetic diversity and population structure and identifying hybrids. Large-scale genetic diversity studies have been conducted on Siamese and saltwater crocodiles [33]. However, universal data on saltwater crocodiles from different geographic origins remain limited because of their wide distribution. The inclusion of diverse populations in genetic studies is crucial to avoid biased allelic frequencies and gene pools, and it also helps to identify true selection signals that overlap across populations owing to the potential impact of genetic drift on genetic diversity. Thousands of SNP_t_ loci were obtained from our DArTseq study, which allowed for the mapping of markers back to the crocodile genomes. Based on sequencing depths of three different datasets (>5X, >10X, and >15X), approximately 17.00–26.00% of the SNP_t_ loci were conserved between Siamese and saltwater crocodile genomes, suggesting the sharing of SNP_t_ loci after their divergence approximately 20 million years ago [5]. By contrast, around 78.00–80.00% of the SNPt loci were specifically diagnostic for Siamese or saltwater crocodile genomes, making them suitable for use as species-diagnostic markers. Our study using DArT markers showed an average *PIC_in_* similar to that of other species, such as the Ring-tailed Dragon (*Ctenophorus caudicinctus*) (0.19) and Plains brown tree frog (*Litoria paraewingi*) (0.18) [34].

In addition, positive *F* values were observed in the Thai populations of Siamese and saltwater crocodiles, which may have been because the specimens were collected from different farms with distinct historical origins as a Wahlund effect [3,35]. This finding differs from that obtained for the Australian population of saltwater crocodiles, where specimens were collected from the wild [14]. Population differentiation (*F*_ST_) was observed among all crocodile populations, notably between CPOTH and CPOAU, even within the same species. This suggests that a substantial genetic exchange or gene flow between the two saltwater crocodile populations did not occur. However, the gene pool of the two saltwater crocodile populations remains consistent in the STRUCTURE analysis. A comparison of the genetic diversity parameters from the SNP analysis and microsatellite genotyping from a previous study [3,4] of Siamese and saltwater crocodile populations in Thailand revealed a tendency toward inbreeding, which was inconsistent with the results of microsatellite genotyping (Table S8). However, the results were consistent with the STRUCTURE analysis of SNPs, which showed only one homozygosity in the gene pool of the Siamese crocodile population, although most crocodiles have been derived from distinct historical origins. This suggests that microsatellite genotyping may provide more informative results in population studies than biallelic SNP genotyping because microsatellites are mutational hotspots that display high levels of polymorphism and a larger number of alleles in diverse populations [36,37,38,39,40]. However, the possibility of false positives resulting from the hierarchical structure generated from the pooling of samples from different captive populations cannot be ruled out. These findings justify the use of different DNA marker methods to support the various objectives and goals of crocodile conservation programs.

### 4.1. Can the Evolutionary Relationships within and between Siamese and Saltwater Crocodiles Be Clarified?

Evidence of the genetic structure of Siamese crocodiles as one group and saltwater crocodiles from Thai and Australian populations as another group was provided via clustering analysis using STUCTURE, DAPC, and PCoA. Species-diagnostic SNPs were detected through molecular testing found for Siamese and saltwater crocodiles, most of which were associated with the methyltransferase, palmitoyl transferase and gonadal family pathway, etc. Similar gene pools were observed in two distantly isolated extant populations of saltwater crocodiles, confirming their genetic differentiation from Siamese crocodiles. The results were consistent with the two species delimitation methods. No indication of selection as a differentiating factor was found; however, the dataset or number of remaining Australian populations in the wild may be too small to detect genetic drift [14]. Thus, these two saltwater crocodile populations may serve as representative populations that are not affected by genetic drift, which is known to cause a decrease in genetic diversity. Diverse populations were analyzed based on the hypothesis that true signals would overlap.

### 4.2. Can Parental and Hybrid Individuals Be Differentiated into Separate Lineages?

Hybridization between Siamese and saltwater crocodiles has previously been detected in the three individuals CSI05, CSI06, and CPO09 using a combination of mtDNA D-loop and microsatellite markers [3,4]. Greater resolution in identifying admixtures and hybrids was achieved in our study by using a larger number of loci, which enabled better sampling of demographic histories and accommodated stochasticity at the levels of polymorphism and lineage sorting [41,42]. The different analytical approaches used for the SNP_t_ data were compared to reliably diagnose the level of hybridization (phylogenetic approach, STRUCTURE, TreeMix, and PCoA) in the presence of the admixture. CSI05 and CSI06 were tentatively identified as F_1_ or F_2_ hybrids, which was consistent with previous microsatellite genotyping results [3,4]. However, CSI05 and CSI06 were identified based on genotypic proportions that exceeded the background noise level (q > 0.05), with q values of 0.007 and 0.009 at sequencing depths of >5X (q values of 0.31 and 0.32 at sequencing depths of >10X and q values of 0.25 and 0.24 at sequencing depths of >15X) observed in the cluster, respectively. Moreover, 36% (31% at sequencing depths of >10X and 25% at sequencing depths of >15X) and 42% (32% at sequencing depths of >10X and 25% at sequencing depths of >15X) of Siamese crocodile-diagnostic SNPs were detected in CSI05 and CSI06, while 64% (69% at sequencing depths of >10X and 75% at sequencing depths of >15X) and 58% (68% at sequencing depths of >10X and 75% at sequencing depths of >15X) of saltwater crocodile-diagnostic SNPs were ascertained. CSI05 and CSI06 both exhibit a predominant saltwater crocodile genetic profile with Siamese crocodile admixture, aligning with the indications from the Structure and Treemix analyses. This suggests that hybridization with potential backcrossing was detected in CSI05 and CSI06 using SNP data; however, the level of hybridization could not be identified based on the TreeMix, PCoA, and phylogenetic tree analyses. Interestingly, CSI05 and CSI06 showed mixed ancestry, with no shared ancestry observed with Siamese crocodiles, even at high *K* values in the Structure analysis. This could potentially be accounted for by the presence of a substantial gene pool within the extensive Siamese crocodile population in Thailand, where the captive population exceeds one million crocodiles [43,44,45]. The retention of divergent lineages or incomplete lineage sorting can be attributed to the significant population size. In addition, individual CPO09 was identified as a saltwater crocodile backcross with a genotypic proportion falling in the upper tail of the q-distribution (q = 0.99) at sequencing depths of >5X (q values of 0.99 at sequencing depths of >10X and >15X), which also possessed a saltwater crocodile mtDNA D-loop haplotype [3,4]. Moreover, 0.04% (0.00% for both sequencing depths of >10X and >15X) of Siamese crocodile-diagnostic SNPs were identified, whereas 99.96% (100% both sequencing depths of >10X and >15X) of saltwater crocodile-diagnostic SNPs were determined in CPO09. This suggests that the multiple backcrossing of saltwater crocodiles may have resulted in CPO09. Several backcross generations, such as BC_1_ and BC_2_, have been suspected in captive crocodile populations in Southeast Asia [3,46]. Crocodilians engage in elaborate courtship and mating rituals, in which female choice is primarily based on the size of male suitors.

Removing suspected hybrids from a collection can protect the genetic integrity of the species, particularly if the collection is used to supply reintroduction candidates or augment the genetic diversity of wild populations. Moreover, removing suspected hybrids can prevent the creation of hybrid swarms, which may have a negative impact on conservation management [47]. Our SNP panel design is a valuable tool for conservation and management, particularly for identifying hybrid individuals and assessing the extent of hybridization events. However, SNP calling on an NGS platform is time-consuming [48]. Compared with previous markers, SNP genotypes can be generated more efficiently using PCR-based species-diagnostic markers, which provide a quicker data turnaround time to better inform management efforts for Siamese crocodiles and allow for more rapid and efficient genetic investigations of these crocodiles.

### 4.3. Enhancing Siamese Crocodile Identification through a Combination of Microsatellite Genotyping, Mitochondrial DNA D-Loop Sequencing, and PCR-Based Species-Diagnostic Markers

The genomic purity of over 1 million Siamese crocodiles residing in crocodile farms throughout Southeast Asia remains controversial and potentially hinders their use for conservation and reintroduction purposes [4,43,44,45]. The genomic purity of captive individuals may have decreased to an unknown degree because of hybridization. The swarming of locally adapted gene pools and their potential contribution to species extinction are serious threats posed by hybridization [43]. Species-diagnostic SNP loci were validated using a PCR-based approach. Of the discovered species-diagnostic SNP loci in Siamese and saltwater crocodiles that were identified, 3 of 20 loci were confirmed to be species-diagnostic. To further confirm the validation tests, an additional 115 specimens, including hybrids and actual species from our previous study [4] (Figure S6), were included in the panel. Several markers that were not validated in all samples showed weak species specificity and may be less reliable as markers for species identification. PCR validation failures are commonly observed in genome SNP bioinformatic analyses, which may be related to the conserved regions in the sequences of both species being adjacent to the species-diagnostic restriction sites. Interestingly, differences in DNA marker patterns between CSI05 and CSI06 were observed using the primer code: CST09-F and R, CPT08-F and R, and CST01-F and R, indicating that CSI05 and CSI06 may differ at hybrid levels. Alternatively, population-diagnostic SNPs may exist in Siamese crocodiles due to genetic drift caused by isolation from different farms. However, the observed difference in hybrid levels between CSI05 and CSI06 was likely due to the highly conserved primer, as demonstrated by extensive DNA marker validation in additional specimens of Siamese and saltwater crocodiles and their hybrids. Another possible explanation is that the absence of a band from CSI05, regardless of which species-diagnostic primer was used, might be associated with the stability of the primer binding site with the primers. The PCR validation step is often observed to fail after DArTseq™ or RADseq bioinformatics analysis, with specific PCR amplification of a representative DArTseq or RAD-seq marker being detected in only some samples, not all, and consequently considered less reliable as species-diagnostic markers [12]. This outcome could be attributed to conserved regions in sequences near species-diagnostic restriction sites in both species.

**Figure 6:**
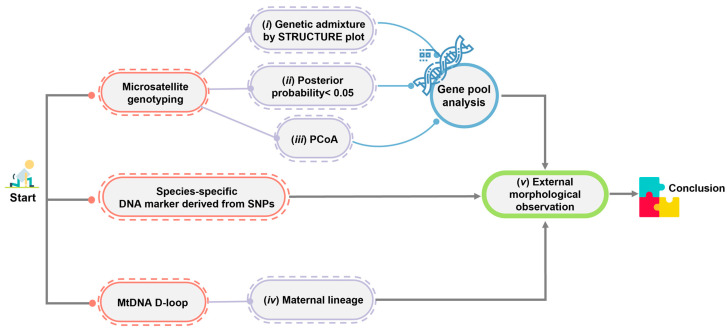
Schematic representation of the criteria used to differentiate Siamese and saltwater crocodiles to demonstrate the hybridization level status.

By combining nuclear microsatellite genotyping, mtDNA D-loop sequencing, and SNPs, the maximum population genomic diversity and minimum human-induced genetic introgression can be ensured among individuals intended for release into the wild. Reliability issues may arise when using genome-wide SNP analyses to identify hybrids when a small number of crocodiles are involved in reintroduction programs [3,4]. However, the discrepancy in the results of the Structure analysis between the genome-wide SNP analyses in this study and the microsatellite genotyping from our previous studies [3,4] might alternatively suggest the need for a different method to identify crocodile species. Sustainable protocols that can identify introgressions and hybridization are urgently required. Combining various genetic markers provides more robust results for identifying historical gene pool patterns of Siamese and saltwater crocodiles. The combination of mitochondrial (maternal inheritance) and nuclear genetic information (Mendelian inheritance), such as microsatellite genotyping and species-diagnostic DNA markers, has proven to be effective in addressing each method’s limitations. The criteria for screening hybrids between Siamese and saltwater crocodiles were extensively revised in this study based on our previous study [4] (Figure 6). Briefly, genetic admixture is assessed at different *K* levels with predictions made by various algorithms, the potential shared gene pool between species is evaluated using a 0.05 threshold for posterior probability at the *K* level, clustering is examined through both PCoA and the STRUCTURE plots to assess specimen groups, and maternal lineage is confirmed via mtDNA D-loop sequences. Species-diagnostic DNA markers can quickly confirm the hybridization status of Siamese crocodile individuals, thereby complementing microsatellite genotyping analysis and aiding in conservation prioritization within a reasonable timeframe before the animal’s release into the wild. However, if clear-cut interpretation cannot be provided by species-diagnostic DNA markers, such as the absence of a DNA band as in CSI05, genome-wide SNP analyses should be conducted to confirm the tested individuals. It is also recommended that Siamese crocodile candidates be genetically screened for reintroduction within each farm to select individuals with high heterozygosity, low relatedness, and low inbreeding coefficients, which will help to improve genetic diversity in the wild. Our study defined five significant guidelines that will directly contribute to the conservation of critically endangered Siamese crocodiles in Thailand: (i) Siamese crocodiles should be donated to reintroduction programs through collaboration with crocodile farms under the TCFA; (ii) our criteria should be applied to detect hybrids and introgression, which remains a major goal for the conservation management of Siamese crocodiles; (iii) individuals from the test panel should be selected for reintroduction programs by collaborative partners; (iv) habitat suitability analysis and habitat improvement of crocodile reintroduction programs should be conducted simultaneously; and (v) solutions should be sought for human–crocodile conflict interactions. Collaboration with national sectors is necessary to monitor the strategy’s long-term effects on Siamese crocodile diversity and embed biodiversity into policy. Such actions will facilitate recovery and protection and enable the evaluation of intervention success.

## 5. Conclusions

This study aimed to determine the impact of hybridization on the differentiation of evolutionary lineages and identify the associated conservation consequences and policy challenges. Through comprehensive sampling and genomic analyses of genome-wide DNA polymorphism data, a complex evolutionary history involving different lineages, evolutionary trajectories, species diversification, and distinct hybrids was identified. SNP panel genotypes have numerous applications in Siamese crocodile conservation management, including the identification of two divergent crocodiles and their hybrid lineages that include critically endangered parental species [3,4]. A criterion for identifying pure species and hybrids was developed by combining previously reported genetic markers (microsatellite genotyping and mtDNA D-loop sequencing) and species-diagnostic DNA markers from SNP loci using multi-genetic approaches [3,4]. This criterion enables the inclusion of authentic Siamese crocodiles in conservation programs. Sustainable genetic integrity relies on precise and accurate data from captive populations, which are essential for ensuring long-term survival through reintroduction programs and in situ/ex situ management.

## Figures and Tables

**Table 1:** Primers are used for the development of species-specific markers.

Primer Name	Locus Id	Sequence 5′–3′	Product Size	A Positive Band on the Target Species
CST09-F	23111606	ACTGCAGACTGAGTGAGTTTC	320 bp	*Crocodylus siamensis*
CST09-R	23111606	CTGTGCCTGAATTGACTGCCA		
CPT08-F	34606790	TTGCCCTTAGCCCCAGTCTA	450 bp	*Crocodylus siamensis*
CPT08-R	34606790	TACTGGGAGGCATCAAACCA		
CST01-F	23113297	AACATTGCTCTGGATGGCCTT	400 bp	*Crocodylus porosus*
CST01-R	23113297	CGGAAATGCCAGCTTTTAGT		

Underlined letters indicate SNPs that can differentiate the target species.

**Table 2:** Genetic diversity among 59 Siamese crocodiles (*Crocodylus siamensis*) and saltwater crocodiles (*Crocodylus porosus*) at sequencing depths of >5X.

Population		N	*N*_a_	*N*_e_	*I*	*H*_o_	*H*_e_	*PIC*	*F*
**CSI ^1^**	Mean	29	1.511	1.095	0.147	0.005	0.079	0.058	0.931
	S.E.	0	0.007	0.001	0.002	0.000	0.001	0.090	0.004
**CPOTH ^2^**	Mean	12	1.511	1.194	0.217	0.020	0.133	0.000	0.907
	S.E.	0	0.007	0.003	0.003	0.002	0.002	0.000	0.005
**CPOAU ^3^**	Mean	18	0.891	0.891	0.000	0.000	0.000	0.000	0.000
	S.E.	0	0.004	0.004	0.000	0.000	0.000	0.000	0.000
**All Population**	Mean	59	1.511	1.051	0.096	0.002	0.046	0.044	0.934
	S.E.	0	0.021	0.001	0.001	0.000	0.001	0.046	0.004

Note: ^1^ Siamese crocodile (*Crocodylus siamensis*), ^2^ saltwater crocodile (*Crocodylus porosus*) from Thailand, ^3^ saltwater crocodile (*Crocodylus porosus*) from Australia.

**Table 3:** Welch’s *t*-test heterozygosity (*H*_o_) and heterozygosity (*H*_e_) of Siamese crocodile (*Crocodylus siamensis*) and saltwater crocodile (*Crocodylus porosus*) at sequencing depths of >5X.

	Population 1	Population 2	df	SE	*t*-Test	*p*-Value
**Observed heterozygosity (*H*_o_)**	CSI ^1^	CPOTH ^2^	0.015	0.002	−7.5	<0.05
	CSI ^1^	CPOAU ^3^	0.005	0	N/A	N/A
	CPOTH ^2^	CPOAU ^3^	0.02	0.002	10	<0.05
**Expected heterozygosity (*H*_e_)**	CSI ^1^	CPOTH ^2^	−0.054	0.002	−24.150	<0.05
	CSI ^1^	CPOAU ^3^	N/A	N/A	N/A	N/A
	CPOTH ^2^	CPOAU ^3^	N/A	N/A	N/A	N/A

Note: ^1^ Siamese crocodile (*Crocodylus siamensis*), ^2^ saltwater crocodile (*Crocodylus porosus*) from Thailand, ^3^ saltwater crocodile (*Crocodylus porosus*) from Australia.

**Table 4:** Comparison of genetic diversity parameters between Siamese crocodile (*Crocodylus siamensis*) and saltwater crocodile (*Crocodylus porosus*) individuals at sequencing depths of >5X.

Population	*H*_o_	*H*_e_	df	*t*-Test	*p*-Value
CSI ^1^	0.005 ± 0.000	0.079 ± 0.001	−0.074	−74	<0.05
CPOTH ^2^	0.020 ± 0.002	0.133 ± 0.002	−0.113	−39.95	<0.05
CPOAU ^3^	0.000 ± 0.000	0.000 ± 0.000	N/A	N/A	N/A

Note: ^1^ Siamese crocodile (*Crocodylus siamensis*), ^2^ saltwater crocodile (*Crocodylus porosus*) from Thailand, ^3^ saltwater crocodile (*Crocodylus porosus*) from Australia.

## Data Availability

The full dataset and metadata from this publication are available in the Dryad Digital Repository. Dataset, https://doi.org/10.5061/dryad.12jm63z35 (https://datadryad.org/stash/share/FwRZQBOYrzjcq4Qsmgq3Oq5jIW4HMpiBYBmDmTPeOWI, accessed on 30 June 2023).

## Supplementary Materials

The following supporting information can be downloaded at: https://www.mdpi.com/article/10.3390/biology12111428/s1, Figure S1: Hamming distance between the Siamese crocodile (*Crocodylus siamensis*, CSI) and saltwater crocodile (*Crocodylus porosus*, CPO) at sequencing depths of >10X. (a) SNP loci with the criterion of species-diagnostic loci (CSI: CPO), and (b) PA loci with the criterion of species-diagnostic loci (CSI: CPO). Sequencing depths of >15X. (c) SNP loci with the criterion of species-diagnostic loci (CSI: CPO), and (d) PA loci with the criterion of species-diagnostic loci (CSI: CPO), Figure S2: (a) Principal coordinate analysis (PCoA) of filtered data of genome-wide single nucleotide polymorphisms (SNP) and presence/absence (PA) among Siamese-saltwater crocodiles, Siamese (CSI)-saltwater (CPOTH) crocodiles (Thai population), and saltwater (CPOAU) crocodiles (Australian population). (b) Discriminant analysis of principal components (DAPC) of among Siamese-saltwater crocodiles, Siamese (CSI)-saltwater (CPOTH) crocodiles (Thai population), and saltwater (CPOAU) crocodiles (Australian population), Figure S3: TreeMix analysis showing the gene flow from population Siamese crocodile (CSI) towards Hybrid crocodile (HB) with genetic drift, Figure S4: Unweighted pair group method with arithmetic mean (UPGMA) phylogeny relationship among Siamese crocodile (*Crocodylus siamensis*), saltwater crocodile (*Crocodylus porosus*), and hybrid crocodile, Figure S5: Homology of putative species-diagnostic loci in Siamese crocodile (*Crocodylus siamensis*) and saltwater crocodile (*Crocodylus porosus*), Figure S6: PCR validation species-diagnostic loci among Siamese crocodile (*Crocodylus siamensis*), saltwater crocodile (*Crocodylus porosus*), and hybrid crocodile. (a) Primer CST09 (locus id: 23111606), (b) primer CST01 (locus id: 23113297), and (d) primer CPT08 (locus id: 34606790). Detailed information on the sampled individuals in Supplementary Table S1, Table S1: Detailed information on the sampled individuals, Table S2: DArT analysis of 59 sampled individual at sequencing depths of >5X, >10X, and 15X, Table S3: Pairwise *F*_ST_ values of Siamese crocodile (*Crocodylus siamensis*) and saltwater crocodile (*Crocodylus porosus*) at sequencing depths of >10X and 15X, Table S4: Genetic diversity among 59 of Siamese crocodile (*Crocodylus siamensis*) and saltwater crocodile (*Crocodylus porosus*) at sequencing depths of >10X and 15X, Table S5: Welch’s *t*-test heterozygosity (*H*_o_) and heterozygosity (*H*_e_) of Siamese crocodile (*Crocodylus siamensis*) and saltwater crocodile (*Crocodylus porosus*) at sequencing depths of >10X and 15X, Table S6: Comparison of genetic diversity parameters between Siamese crocodile (*Crocodylus siamensis*) and saltwater crocodile (*Crocodylus porosus*) individuals at sequencing depths of >10X and 15X, Table S7: Proportion of conserved SNPt loci in Siamese and saltwater crocodile populations at sequencing depths of >5X, >10X and 15X., Table S8: Genetic diversity among Siamese crocodile (*Crocodylus siamensis*) and saltwater crocodile (*Crocodylus porosus*).
