# Supplementary material for: Preserving Pure Siamese Crocodile Populations: A Comprehensive Approach Using Multi-Genetic Tools
Source: Biology (Basel). 2023 Nov 13;12(11):1428. doi: 10.3390/biology12111428 (PMC10669835; doi:10.3390/biology12111428)
Supplement: Supplementary file 1 [file biology-12-01428-s001.zip › biology-2684183-supplementary.pdf]

Table S1. Detailed information on the sampled individuals.

No.	Abbreviation/Code	Species	Captive/Wild population	Locality	Reference
1	CSI01	*Crocodylus siamensis*	Captivity # 1	Thailand	Lapbenjakul et al., 2017
2	CSI02	*Crocodylus siamensis*	Captivity # 1	Thailand	Lapbenjakul et al., 2017
3	CSI03	*Crocodylus siamensis*	Captivity # 1	Thailand	Lapbenjakul et al., 2017
4	CSI04	*Crocodylus siamensis*	Captivity # 1	Thailand	Lapbenjakul et al., 2017
5	CSI05	*Crocodylus siamensis*	Captivity # 1	Thailand	Lapbenjakul et al., 2017
6	CSI06	*Crocodylus siamensis*	Captivity # 1	Thailand	Lapbenjakul et al., 2017
7	CSI07	*Crocodylus siamensis*	Captivity # 2	Thailand	Lapbenjakul et al., 2017
8	CSI09	*Crocodylus siamensis*	Captivity # 2	Thailand	Lapbenjakul et al., 2017
9	CSI10	*Crocodylus siamensis*	Captivity # 3	Thailand	Lapbenjakul et al., 2017
10	CSI11	*Crocodylus siamensis*	Captivity # 3	Thailand	Lapbenjakul et al., 2017
11	CSI12	*Crocodylus siamensis*	Captivity # 3	Thailand	Lapbenjakul et al., 2017
12	CSI13	*Crocodylus siamensis*	Captivity # 3	Thailand	Lapbenjakul et al., 2017
13	CSI14	*Crocodylus siamensis*	Captivity # 4	Thailand	Lapbenjakul et al., 2017
14	CSI15	*Crocodylus siamensis*	Captivity # 4	Thailand	Lapbenjakul et al., 2017
15	CSI20	*Crocodylus siamensis*	Captivity # 5	Thailand	Lapbenjakul et al., 2017
16	CSI21	*Crocodylus siamensis*	Captivity # 5	Thailand	Lapbenjakul et al., 2017
17	CSI22	*Crocodylus siamensis*	Captivity # 6	Thailand	Lapbenjakul et al., 2017
18	CSI23	*Crocodylus siamensis*	Captivity # 6	Thailand	Lapbenjakul et al., 2017
19	CSI24	*Crocodylus siamensis*	Captivity # 6	Thailand	Lapbenjakul et al., 2017
20	CSI25	*Crocodylus siamensis*	Captivity # 6	Thailand	Lapbenjakul et al., 2017
21	CSI27	*Crocodylus siamensis*	Captivity # 7	Thailand	Lapbenjakul et al., 2017
22	CSI28	*Crocodylus siamensis*	Captivity # 7	Thailand	Lapbenjakul et al., 2017
23	CSI29	*Crocodylus siamensis*	Captivity # 8	Thailand	Lapbenjakul et al., 2017
24	CSI30	*Crocodylus siamensis*	Captivity # 8	Thailand	Lapbenjakul et al., 2017
25	CSI34	*Crocodylus siamensis*	Captivity # 9	Thailand	Lapbenjakul et al., 2017
26	CSI35	*Crocodylus siamensis*	Captivity # 9	Thailand	Lapbenjakul et al., 2017
27	CSI36	*Crocodylus siamensis*	Captivity # 9	Thailand	Lapbenjakul et al., 2017
28	CSI37	*Crocodylus siamensis*	Captivity # 10	Thailand	Lapbenjakul et al., 2017
29	CSI38	*Crocodylus siamensis*	Captivity # 10	Thailand	Lapbenjakul et al., 2017
30	CPOTH01	*Crocodylus porosus*	Captivity # 2	Thailand	Lapbenjakul et al., 2017

No.	Abbreviation/Code	Species	Captive/Wild population	Locality	Reference
31	CPOTH02	*Crocodylus porosus*	Captivity # 2	Thailand	Lapbenjakul et al., 2017
32	CPOTH03	*Crocodylus porosus*	Captivity # 2	Thailand	Lapbenjakul et al., 2017
33	CPOTH04	*Crocodylus porosus*	Captivity # 2	Thailand	Lapbenjakul et al., 2017
34	CPOTH05	*Crocodylus porosus*	Captivity # 3	Thailand	Lapbenjakul et al., 2017
35	CPOTH08	*Crocodylus porosus*	Captivity # 3	Thailand	Lapbenjakul et al., 2017
36	CPOTH09	*Crocodylus porosus*	Captivity # 5	Thailand	Lapbenjakul et al., 2017
37	CPOTH11	*Crocodylus porosus*	Captivity # 6	Thailand	Lapbenjakul et al., 2017
38	CPOTH12	*Crocodylus porosus*	Captivity # 6	Thailand	Lapbenjakul et al., 2017
39	CPOTH15	*Crocodylus porosus*	Captivity # 9	Thailand	Lapbenjakul et al., 2017
40	CPOTH16	*Crocodylus porosus*	Captivity # 9	Thailand	Lapbenjakul et al., 2017
41	CPOTH17	*Crocodylus porosus*	Captivity # 9	Thailand	Lapbenjakul et al., 2017
42	CPOAU0384	*Crocodylus porosus*	Wild	Australia	Fukuda et al., 2022
43	CPOAU0493	*Crocodylus porosus*	Wild	Australia	Fukuda et al., 2022
44	CPOAU0392	*Crocodylus porosus*	Wild	Australia	Fukuda et al., 2022
45	CPOAU0494	*Crocodylus porosus*	Wild	Australia	Fukuda et al., 2022
46	CPOAU0495	*Crocodylus porosus*	Wild	Australia	Fukuda et al., 2022
47	CPOAU0497	*Crocodylus porosus*	Wild	Australia	Fukuda et al., 2022
48	CPOAU0753	*Crocodylus porosus*	Wild	Australia	Fukuda et al., 2022
49	CPOAU0698	*Crocodylus porosus*	Wild	Australia	Fukuda et al., 2022
50	CPOAU0675	*Crocodylus porosus*	Wild	Australia	Fukuda et al., 2022
51	CPOAU0683	*Crocodylus porosus*	Wild	Australia	Fukuda et al., 2022
52	CPOAU0712	*Crocodylus porosus*	Wild	Australia	Fukuda et al., 2022
53	CPOAU0641	*Crocodylus porosus*	Wild	Australia	Fukuda et al., 2022
54	CPOAU0737	*Crocodylus porosus*	Wild	Australia	Fukuda et al., 2022
55	CPOAU0746	*Crocodylus porosus*	Wild	Australia	Fukuda et al., 2022
56	CPOAU0661	*Crocodylus porosus*	Wild	Australia	Fukuda et al., 2022
57	CPOAU0693	*Crocodylus porosus*	Wild	Australia	Fukuda et al., 2022
58	CPOAU0695	*Crocodylus porosus*	Wild	Australia	Fukuda et al., 2022
59	CPOAU0722	*Crocodylus porosus*	Wild	Australia	Fukuda et al., 2022
60	CB01	*Crocodylus siamensis*	–	Thailand	Ariyaraphong et al., 2023
61	CB02	*Crocodylus siamensis*	–	Thailand	Ariyaraphong et al., 2023
62	CB03	*Crocodylus siamensis*	–	Thailand	Ariyaraphong et al., 2023
63	CB04	*Crocodylus siamensis*	–	Thailand	Ariyaraphong et al., 2023

No.	Abbreviation/Code	Species	Captive/Wild population	Locality	Reference
64	CB05	*Crocodylus siamensis*	–	Thailand	Ariyaraphong et al., 2023
65	CB11	*Crocodylus siamensis*	–	Thailand	Ariyaraphong et al., 2023
66	CB27	*Crocodylus siamensis*	–	Thailand	Ariyaraphong et al., 2023
67	CP01	*Crocodylus porosus*	–	Thailand	Ariyaraphong et al., 2023
68	CP02	*Crocodylus porosus*	–	Thailand	Ariyaraphong et al., 2023
69	CP03	*Crocodylus porosus*	–	Thailand	Ariyaraphong et al., 2023
70	CP04	*Crocodylus porosus*	–	Thailand	Ariyaraphong et al., 2023
71	CP05	*Crocodylus porosus*	–	Thailand	Ariyaraphong et al., 2023
72	CP06	*Crocodylus porosus*	–	Thailand	Ariyaraphong et al., 2023
73	CP11	*Crocodylus porosus*	–	Thailand	Ariyaraphong et al., 2023
74	CP14	*Crocodylus porosus*	–	Thailand	Ariyaraphong et al., 2023
75	CP16	*Crocodylus porosus*	–	Thailand	Ariyaraphong et al., 2023
76	CP17	*Crocodylus porosus*	–	Thailand	Ariyaraphong et al., 2023

Table S2. DArT analysis of 59 sampled individuals at sequencing depths of >5X, >10X, and 15X.#

		Species-diagnostic loci	
		**SNP**	**PA**
**Sequencing depths of >5x**	Total number of DArT analyses	53,160	28,775
	Species-diagnostic loci	5,091	4,248
	Overall mean distance between Siamese crocodile and saltwater crocodile	1.00±0.000	1.00±0.000
	Overall mean distance within Siamese crocodile	0.520 ± 0.043	0.388 ± 0.029
	Overall mean distance within saltwater crocodile	0.710 ± 0.020	0.667 ± 0.026
	CATT test	$\chi2$ = 56.00–118.00 $p < 0.001$	$\chi2$ = 56.00–59.00 $p < 0.001$
**Sequencing depths of >10x**	Total number of DArT analyses	53,160	28,775
	Species-diagnostic loci	1,247	324
	Overall mean distance between Siamese crocodile and saltwater crocodile	1.00±0.00	1.00±0.000
	Overall mean distance within Siamese crocodile	0.510 ± 0.033	0.300 ± 0.029
	Overall mean distance within saltwater crocodile	0.690 ± 0.020	0.657 ± 0.026
	CATT test	$\chi2$ = 56.00–117.00 $p < 0.001$	$\chi2$ = 56.00–58.00 $p < 0.001$
**Sequencing depths of >15x**	Total number of DArT analyses	53,160	28,775
	Species-diagnostic loci	193	16
	Overall mean distance between Siamese crocodile and saltwater crocodile	1.00±0.00	1.00±0.00
	Overall mean distance within Siamese crocodile	0.500 ± 0.033	0.300 ± 0.028
	Overall mean distance within saltwater crocodile	0.68 ± 0.020	0.655 ± 0.026
	CATT test	$\chi2$ = 56.00–115.00 $p < 0.001$	$\chi2$ = 55.00 $p < 0.001$

Table S3. Pairwise F_{ST} values of Siamese crocodiles (*Crocodylus siamensis*) and saltwater crocodiles (*Crocodylus porosus*) at sequencing depths of >5X, >10X, and 15X.

**Sequencing depths**	**Population**	**Population**	F_{ST}	***p*-value**
**> 5x**	CPOAU[1]	CPOTH[2]	0.219	<0.05
	CPOAU	CSI[3]	1.000	<0.05
	CPOTH	CSI	0.842	<0.05
**> 10x**	CPOAU[1]	CPOTH[2]	0.211	<0.05
	CPOAU	CSI[3]	1.000	<0.05
	CPOTH	CSI	0.942	<0.05
**> 15x**	CPOAU[1]	CPOTH[2]	0.209	<0.05
	CPOAU	CSI[3]	1.000	<0.05
	CPOTH	CSI	0.954	<0.05

Note: [1]saltwater crocodile (*Crocodylus porosus*) from Australia, [2]saltwater crocodile (*Crocodylus porosus*) from Thailand, [3]Siamese crocodile (*Crocodylus siamensis*).

Table S4. Genetic diversity among 59 Siamese crocodiles (*Crocodylus siamensis*) and saltwater crocodiles (*Crocodylus porosus*) at sequencing depths of >10X and 15X.

**Sequencing depths**	**Population**		**N**	N_a	N_e	I	H_o	H_e	*PIC*	F
**> 10x**	CSI [1]	Mean	29	1.511	1.095	0.145	0.005	0.079	0.058	0.931
		S.E.	0	0.007	0.001	0.002	0	0.001	0.09	0.004
	CPOTH [2]	Mean	12	1.511	1.194	0.216	0.02	0.133	0	0.907
		S.E.	0	0.007	0.003	0.003	0.002	0.002	0	0.005
	CPOAU [3]	Mean	18	0.89	0.891	0	0	0	0	0
		S.E.	0	0.004	0.004	0	0	0	0	0
	All Population	Mean	59	1.304	1.051	0.120	0.002	0.046	0.044	0.934
		S.E.	0	0.021	0.001	0.001	0	0.001	0.046	0.004
**> 15x**	CSI [1]	Mean	29	1.511	1.095	0.145	0.005	0.079	0.058	0.931
		S.E.	0	0.007	0.001	0.002	0	0.001	0.09	0.004
	CPOTH [2]	Mean	12	1.511	1.193	0.216	0.02	0.133	0	0.907
		S.E.	0	0.007	0.003	0.003	0.002	0.002	0	0.005
	CPOAU [3]	Mean	18	0.890	0.891	0	0	0	0	0
		S.E.	0	0.004	0.004	0	0	0	0	0
	All Population	Mean	59	1.304	1.060	0.120	0.002	0.046	0.044	0.934
		S.E.	0	0.021	0.001	0.001	0	0.001	0.046	0.004

Note: [1]saltwater crocodile (*Crocodylus porosus*) from Australia, [2]saltwater crocodile (*Crocodylus porosus*) from Thailand, [3]Siamese crocodile (*Crocodylus siamensis*).

Table S5. Welch's *t*-test heterozygosity (H_o) and heterozygosity (H_e) of Siamese crocodiles (*Crocodylus siamensis*) and saltwater crocodiles (*Crocodylus porosus*) at sequencing depths of >10X and 15X.

**Sequencing depths**	**Population**	H_o	H_e	**df**	**t-test**	***p*-value**
**> 10x**	CSI[1]	0.005±0.000	0.079±0.001	–0.074	–74	<0.05
	CPOTH[2]	0.020±0.002	0.133±0.002	–0.113	–39.95	<0.05
	CPOAU[3]	0.000±0.000	0.000±0.000	N/A	N/A	N/A
**> 15x**	CSI[1]	0.005±0.000	0.079±0.001	–0.071	–72	<0.05
	CPOTH[2]	0.020±0.002	0.133±0.002	–0.112	–39.94	<0.05
	CPOAU[3]	0.000±0.000	0.000±0.000	N/A	N/A	N/A

Note: [1]saltwater crocodile (*Crocodylus porosus*) from Australia, [2]saltwater crocodile (*Crocodylus porosus*) from Thailand, [3]Siamese crocodile (*Crocodylus siamensis*).

Table S6. Comparison of genetic diversity parameters between Siamese crocodile (*Crocodylus siamensis*) and saltwater crocodile (*Crocodylus porosus*) individuals at sequencing depths of >10X and 15X.

Sequencing depths		Population 1	Population 2	df	SE	t-test	p-value
**> 10x**	Observed	CSI[1]	CPOTH[2]	0.015	0.002	-7.5	<0.05
	heterozygosity	CSI[1]	CPOAU[3]	0.005	0	N/A	N/A
	(H_o)	CPOTH[2]	CPOAU[3]	0.02	0.002	10	<0.05
	Expected	CSI[1]	CPOTH[2]	-0.054	0.001	-24.149	<0.05
	heterozygosity	CSI[1]	CPOAU[3]	N/A	N/A	N/A	N/A
	(H_e)	CPOTH[2]	CPOAU[3]	N/A	N/A	N/A	N/A
**> 15x**	Observed	CSI[1]	CPOTH[2]	0.015	0.002	-7.5	<0.05
	heterozygosity	CSI[1]	CPOAU[3]	0.005	0	N/A	N/A
	(H_o)	CPOTH[2]	CPOAU[3]	0.02	0.002	10	<0.05
	Expected	CSI[1]	CPOTH[2]	-0.054	0.001	-24.149	<0.05
	heterozygosity	CSI[1]	CPOAU[3]	N/A	N/A	N/A	N/A
	(H_e)	CPOTH[2]	CPOAU[3]	N/A	N/A	N/A	N/A

Note: [1]saltwater crocodile (*Crocodylus porosus*) from Australia, [2]saltwater crocodile (*Crocodylus porosus*) from Thailand, [3]Siamese crocodile (*Crocodylus siamensis*).

Table S7. Proportion of conserved SNP_t loci in Siamese and saltwater crocodile populations at sequencing depths of >5X, >10X, and 15X.

Sequencing depths	Siamese and saltwater crocodiles	Within the Siamese crocodile population	Within saltwater crocodiles
**> 5x**	25.80%	79.80%	78.30%
**> 10x**	43.60%	100.00%	99.99%
**> 15x**	44.70%	100.00%	100.00%

Table S8. Genetic diversity among Siamese crocodiles (*Crocodylus siamensis*) and saltwater crocodiles (*Crocodylus porosus*).

**Population**		**SNP data**			**Microsatellite data** [1]		
		H_o	H_e	*PIC*	H_o	H_e	*PIC*
CSI[2]	Mean	0.005	0.079	0.058	0.483	0.583	0.556
	S.E.	0.000	0.001	0.090	0.067	0.068	0.321
CPOTH[3]	Mean	0.020	0.133	0.000	0.598	0.671	0.637
	S.E.	0.002	0.002	0.000	0.058	0.044	0.212
CPOAU[4]	Mean	0.000	0.000	0.000	-	-	-
	S.E.	0.000	0.000	0.000	-	-	-
All Population	Mean	0.002	0.046	0.044	0.541	0.627	0.697
	S.E.	0.000	0.001	0.046	0.045	0.041	0.201

Note: [1]Data were from retrieved Lapbenjakul et al. (2017), [2]Siamese crocodile (*Crocodylus siamensis*), [3]saltwater crocodile (*Crocodylus porosus*) from Thailand, [4]saltwater crocodile (*Crocodylus porosus*) from Australia (Fukuda et al., 2022)1

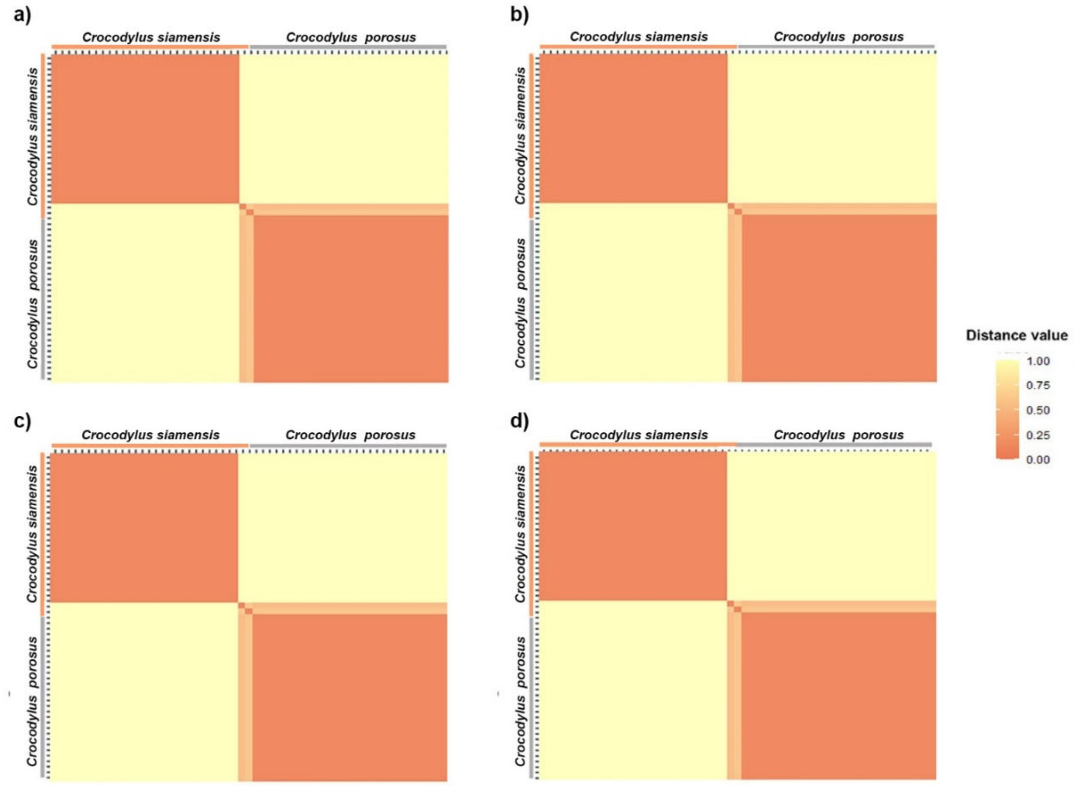

Figure S1. Hamming distance between the Siamese crocodile (*Crocodylus siamensis*, CSI) and saltwater crocodile (*Crocodylus porosus*, CPO) at sequencing depths of >10X. (a) SNP loci with the criterion of species-diagnostic loci (CSI: CPO), and (b) PA loci with the criterion of species-diagnostic loci (CSI: CPO). Sequencing depths of >15X. (c) SNP loci with the criterion of species-diagnostic loci (CSI: CPO), and (d) PA loci with the criterion of species-diagnostic loci (CSI: CPO)

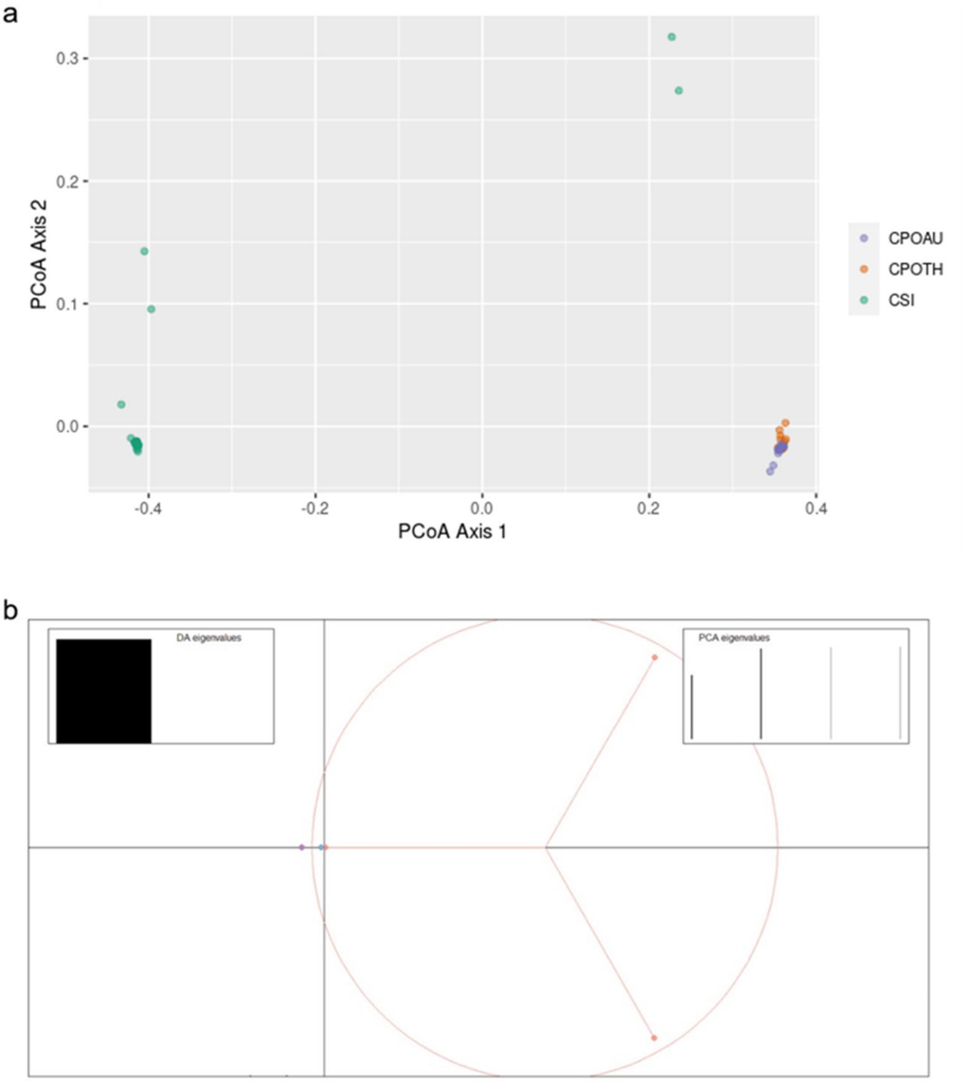

Figure S2. (a) Principal coordinate analysis (PCoA) of filtered data of genome-wide single-nucleotide polymorphisms (SNP) among Siamese–saltwater crocodiles, Siamese (CSI)–saltwater (CPOTH) crocodiles (Thai population), and saltwater (CPOAU) crocodiles (Australian population). (b) Discriminant analysis of principal components (DAPC) of among Siamese–saltwater crocodiles, Siamese (CSI)–saltwater (CPOTH) crocodiles (Thai population), and saltwater (CPOAU) crocodiles (Australian population).

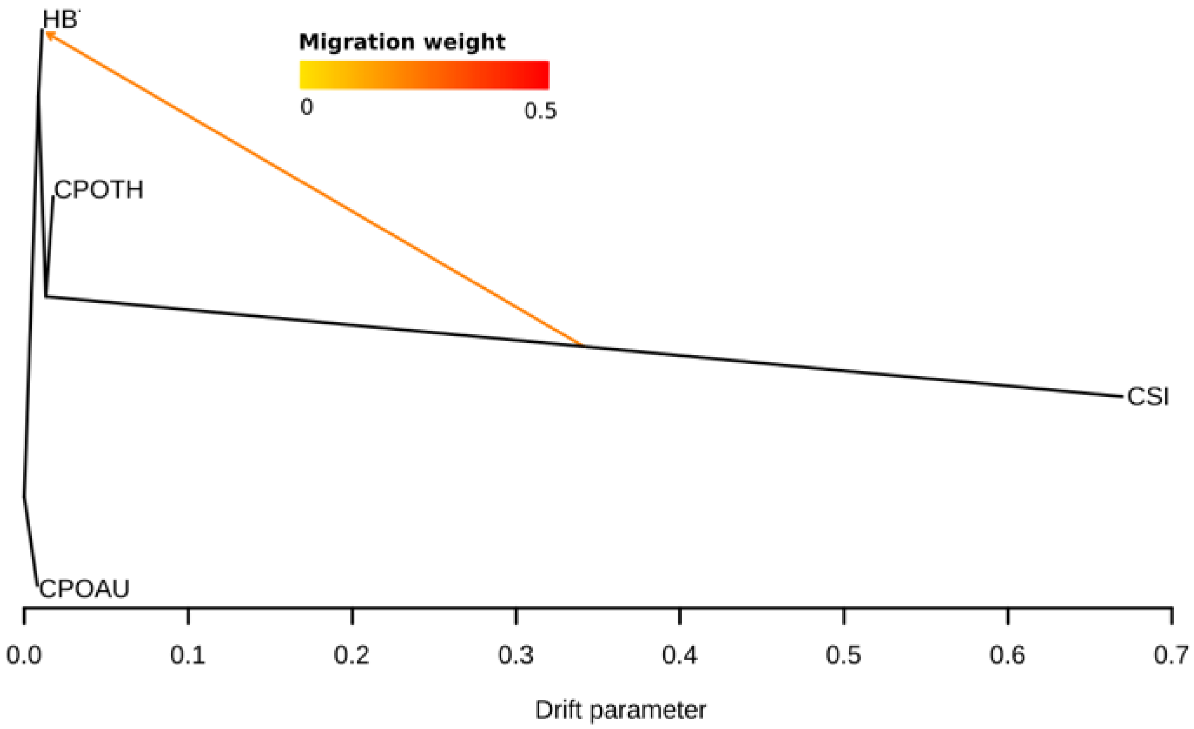

Figure S3. TreeMix analysis showing the gene flow from population Siamese crocodile (CSI) towards Hybrid crocodile (HB) with genetic drift.

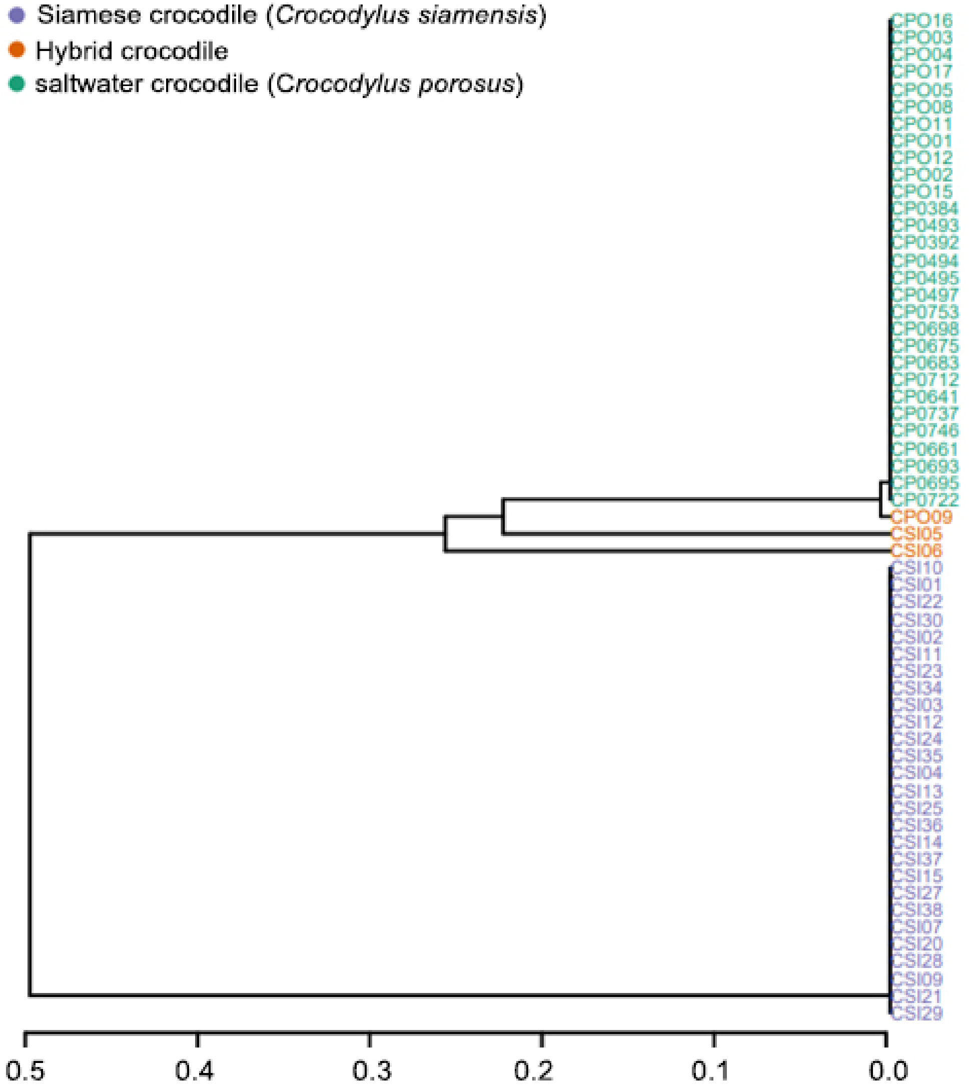

Figure S4. Unweighted pair group method with arithmetic mean (UPGMA) phylogeny relationship among Siamese crocodile (*Crocodylus siamensis*), saltwater crocodile (*Crocodylus porosus*), and hybrid crocodile.

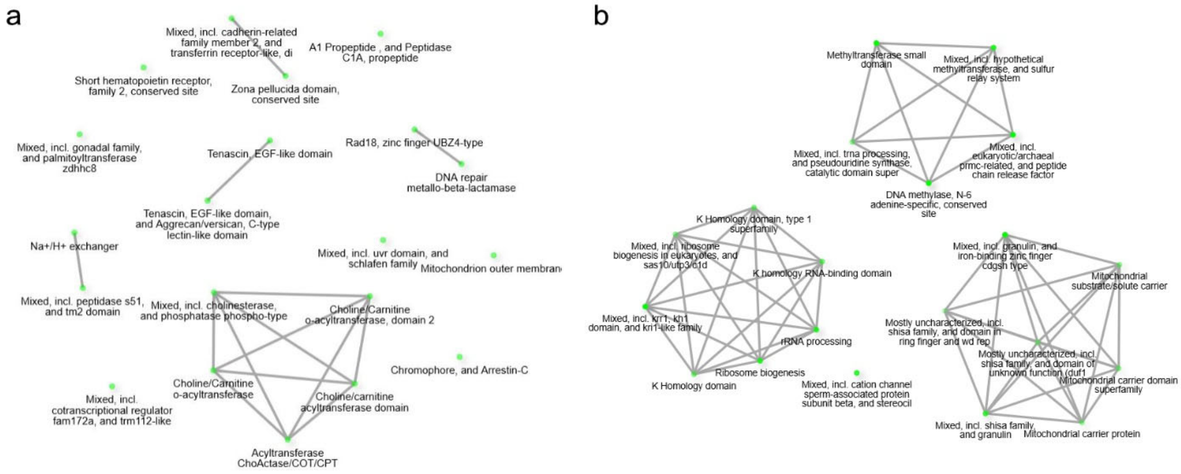

Figure S5. Homology of putative species-specific loci in Siamese crocodile (*Crocodylus siamensis*) and saltwater crocodile (*Crocodylus porosus*).

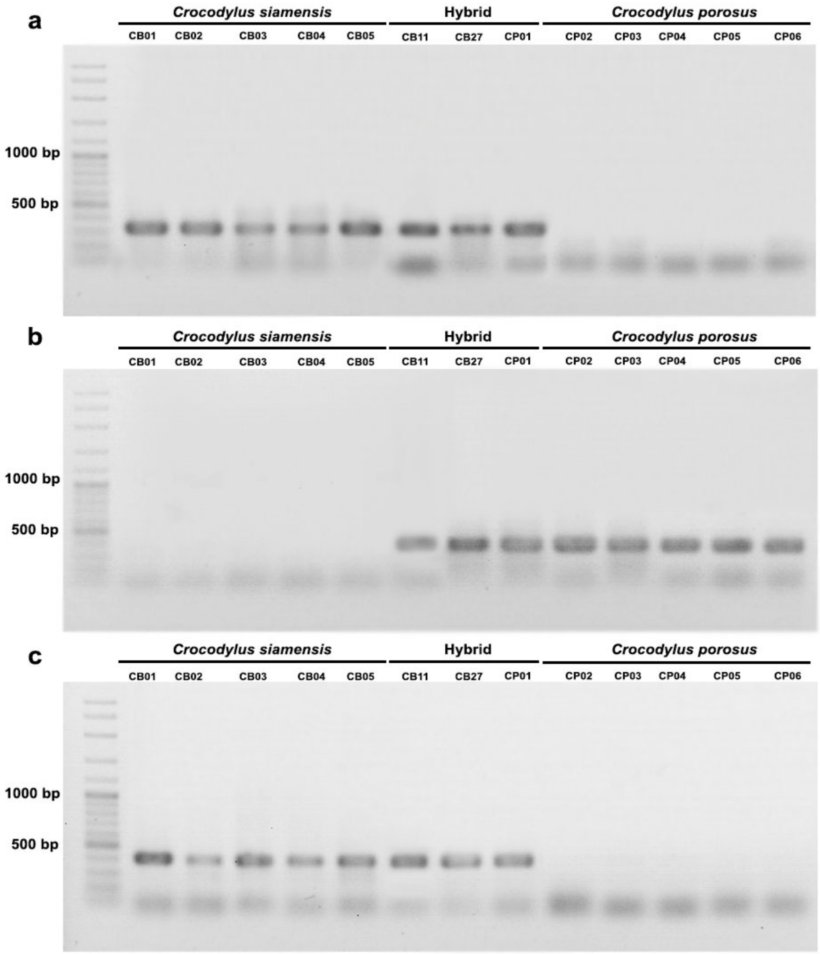

Figure S6. PCR validation species-diagnostic loci among Siamese crocodile (*Crocodylus siamensis*), saltwater crocodile (*Crocodylus porosus*), and hybrid crocodile. (a) Primer CST09 (locus id: 23111606), (b) primer CST01 (locus id: 23113297), and (d) primer CPT08 (locus id: 34606790). Detailed information on the sampled individuals in Supplementary Table S1
